# Regional Distributions of Iron, Copper and Zinc and Their Relationships With Glia in a Normal Aging Mouse Model

**DOI:** 10.3389/fnagi.2019.00351

**Published:** 2019-12-19

**Authors:** Azhaar Ashraf, Christos Michaelides, Thomas A. Walker, Antigoni Ekonomou, Maria Suessmilch, Achvini Sriskanthanathan, Semhar Abraha, Adam Parkes, Harold G. Parkes, Kalotina Geraki, Po-Wah So

**Affiliations:** ^1^Department of Neuroimaging, Institute of Psychiatry, Psychology, and Neuroscience, King’s College London, London, United Kingdom; ^2^Diamond Light Source, Harwell Science and Innovation Campus, Didcot, United Kingdom

**Keywords:** synchrotron radiation-based X-ray fluorescence elemental mapping, microglia, astrocytes, iron, copper, zinc, aging, glial dystrophy

## Abstract

Microglia and astrocytes can quench metal toxicity to maintain tissue homeostasis, but with age, increasing glial dystrophy alongside metal dyshomeostasis may predispose the aged brain to acquire neurodegenerative diseases. The aim of the present study was to investigate age-related changes in brain metal deposition along with glial distribution in normal C57Bl/6J mice aged 2-, 6-, 19- and 27-months (*n* = 4/age). Using synchrotron-based X-ray fluorescence elemental mapping, we demonstrated age-related increases in iron, copper, and zinc in the basal ganglia (*p* < 0.05). Qualitative assessments revealed age-associated increases in iron, particularly in the basal ganglia and zinc in the white matter tracts, while copper showed overt enrichment in the choroid plexus/ventricles. Immunohistochemical staining showed augmented numbers of microglia and astrocytes, as a function of aging, in the basal ganglia (*p* < 0.05). Moreover, qualitative analysis of the glial immunostaining at the level of the fimbria and ventral commissure, revealed increments in the number of microglia but decrements in astroglia, in older aged mice. Upon morphological evaluation, aged microglia and astroglia displayed enlarged soma and thickened processes, reminiscent of dystrophy. Since glial cells have major roles in metal metabolism, we performed linear regression analysis and found a positive association between iron (*R*^2^ = 0.57, *p* = 0.0008), copper (*R*^2^ = 0.43, *p* = 0.0057), and zinc (*R*^2^ = 0.37, *p* = 0.0132) with microglia in the basal ganglia. Also, higher levels of iron (*R*^2^ = 0.49, *p* = 0.0025) and zinc (*R*^2^ = 0.27, *p* = 0.040) were correlated to higher astroglia numbers. Aging was accompanied by a dissociation between metal and glial levels, as we found through the formulation of metal to glia ratios, with regions of basal ganglia being differentially affected. For example, iron to astroglia ratio showed age-related increases in the substantia nigra and globus pallidus, while the ratio was decreased in the striatum. Meanwhile, copper and zinc to astroglia ratios showed a similar regional decline. Our findings suggest that inflammation at the choroid plexus, part of the blood-cerebrospinal-fluid barrier, prompts accumulation of, particularly, copper and iron in the ventricles, implying a compromised barrier system. Moreover, age-related glial dystrophy/senescence appears to disrupt metal homeostasis, likely due to induced oxidative stress, and hence increase the risk of neurodegenerative diseases.

## Introduction

Trace metals are essential for biochemical and physiological processes as components of various vitamins, enzymes, and cofactors (Li et al., 2017). Iron is present in copious amounts in the brain, performing pleiotropic functions including neurotransmitter synthesis, neuronal myelination and adenosine triphosphate (ATP) synthesis (Ashraf et al., 2018). Other metals such as copper, zinc and calcium are required alongside iron for modulation of synaptic activity and neuronal plasticity (Popescu and Nichol, 2011; Wang et al., 2012; Braidy et al., 2017; Li et al., 2017). Their balance within the brain is regulated in a complex fashion by brain-barrier systems (blood-brain-barrier, BBB; blood-cerebrospinal fluid-barrier, BCSFB) and glial cells in the CNS milieu. Also, glial cells, i.e., microglia and astrocytes, can sequester metals to protect neurons from metal-induced toxicity (Bishop et al., 2011). Astrocytes appear to have a central role in attenuating neural excitotoxicity by scavenging metals that cross brain-barrier defenses, with microglia also partaking in the immunity against metal accumulations (Zheng et al., 2010; Morales et al., 2017).

Since aging is characterized by perturbed permeability of brain-barriers and glial dystrophy/senescence, brain metal concentration, which would otherwise be kept within narrow limits, becomes dysregulated (Bishop et al., 2011; Popescu and Nichol, 2011; Rathore et al., 2012; Braidy et al., 2017). The altered compositions of trace metals may induce oxidative stress and contribute to advanced age, being the major risk factor for neurodegenerative diseases including Alzheimer’s and Parkinson’s disease (PD; Popescu et al., 2009; Popescu and Nichol, 2011; Grochowski et al., 2019). The analysis of metal content, in terms of their age-related accumulation in different brain regions, may be useful for monitoring the changes accompanying normal and abnormal aging, and provide avenues for maintaining optimal brain health. While there have been many reports indicating brain iron increases with aging between different regions, some have used bulk measurements of tissue samples from different areas rather than by spatial iron mapping (Markesbery et al., 1984); others by histochemical Perl’s staining which is not quantitative (Connor et al., 1990; Benkovic and Connor, 1993; Burdo et al., 1999); and some are based on relaxometry or quantitative susceptibility mapping MRI measurements which indirectly assessed iron by its effects on the surrounding protons (Langkammer et al., 2012). Most notably, none to our knowledge have spatially mapped transition metals at reasonably high resolutions throughout the life-course of a normally aging organism. Therefore, the aim of this study was to examine brain metal levels using synchrotron-radiation X-ray fluorescence (SRXRF) elemental mapping over the normal aging life-course of C57Bl/6J mice at 2-, 6-, 19- and 27-months, corresponding to “post-adolescence, young adults, elderly and very elderly,” respectively. Assessment of microglia and astrocytes by ionized calcium binding adaptor molecule 1 (Iba1) and glial fibrillary acidic protein (GFAP) immunohistochemistry (IHC), respectively, was also performed. We have focussed on the basal ganglia which we and others have demonstrated to accumulate iron with aging (Aquino et al., 2009; Walker et al., 2016). We have previously reported the quantitative assessments of iron; and ferritin-, Iba1- and GFAP-immunopositive cells in the basal ganglia at these ages (Walker et al., 2016). Here, we have extended the study/re-analyzed the data to include both qualitative and quantitative assessments of other metals, and particularly focussed on investigating the relationships between metals and ferritin-, Iba1- and GFAP-immunopositive cells in the basal ganglia. Additionally, metal contents from the ventral hippocampus and the cingulate cortex are also included in this study, as these regions are routinely affected in normal aging and neurodegenerative diseases.

## Materials and Methods

### Animals

Male C57BL/6J mouse brains were obtained from Shared Aging Research Models (ShARM, Sheffield, UK). Mice were culled at 2-, 6-, 19- and 27-months of age (*n* = 4/age) by rising CO_2_ inhalation. Ages of mice chosen for study were comparable to different stages of life from just post-adolescence, adult, midlife, elderly and the very elderly. The heads were removed from the body and heads fixed in 4% paraformaldehyde for 1 week. The brains were then dissected out of the skulls and brains stored in phosphate-buffered saline (PBS, 4°C) with 0.05% sodium azide prior to cryosectioning for SRXRF and IHC (see below). Ethical approval was not required for this study according to the Animals (Scientific Procedures) Act 1986 (ASPA).

### Brain Cryosectioning

Brains were cryoprotected in 30% sucrose in PBS with 0.05% sodium azide and then cryosectioned coronally to produce 40 μm thick frozen sections that were mounted onto 4 μm thick Ultralene film (Spex Sample-Prep, NJ, USA) secured to a customized holder for SRXRF. Adjacent 20 μm thick cryosections sections were also obtained and mounted onto Superfrost plus microscope slides for IHC.

### SRXRF Elemental Mapping and Analysis

SRXRF of the whole/right hemisphere of brain tissue sections was performed at the Diamond Light Source synchrotron radiation facility (microfocus beamline I18; Didcot, UK). Brain sections were mounted at a 45° angle with respect to the incoming X-ray beam and the detector to minimize scatter contribution and scanned raster fashion using a beam with a diameter (resolution) of 100 μm and 11 keV energy. Full energy dispersive spectra were collected for each sample point exposed to the beam, fitted, and the net peak areas of the characteristic peaks of iron, zinc, and copper (Supplementary Table S1) were evaluated using PyMca (Solé et al., 2007). The photon flux on the samples, required for quantification was estimated by measurement of a reference metal film (AXO, Dresden, GmbH). SRXRF elemental maps of pixel-by-pixel elemental metal concentrations (parts per million, ppm) were obtained. Regions of interest (ROIs) were placed on the elemental metal maps to obtain average concentrations in brain regions: substantia nigra (Bregma −3.08 to −3.64 mm), globus pallidus (Bregma −0.22 to −0.70 mm), striatum (Bregma +1.10 mm to +0.14), cingulate cortex (Bregma +1.10 to +0.26 mm) and ventral hippocampus (Bregma −2.80 to −3.52 mm).

### Immunohistochemistry

Standard diaminobenzidine (DAB)-IHC was performed. Firstly, endogenous peroxidase activity was blocked by incubation with 1% hydrogen peroxide in tris-buffered saline (pH 7.4) containing 0.2% Triton X-100 (TBS+; 30 mins, ambient temperature). Following 5 mins × 2 washes in TBS+, non-specific binding was blocked using 10% skimmed milk powder (SMP) in TBS+ (2 h, ambient temperature). Then the sections were incubated with either rabbit anti-ferritin polyclonal antibody (F6136, against ferritin light and heavy chains; Sigma-Aldrich, Poole, Dorset, UK), rabbit polyclonal anti-Iba1 antibody (019–19741; Wako Pure Chemical Industries, Richmond, VA, USA), or rabbit polyclonal anti-GFAP antibody (DAKO, High Wycombe, UK) in 5% SMP in TBS+ (overnight, 4°C). After washes in TBS+, slides were incubated with secondary biotinylated anti-rabbit IgG (1:200, DAKO) in 5% SMP in TBS+ (2 h, ambient temperature). The avidin-biotin binding was then performed using the ELITE ABC kit (Vector, Peterborough, UK) prior to the chromogenic reaction using the Impact DAB peroxidase kit (Vector). Finally, after dehydration in ascending grades of ethanol, slides were cleared in xylene and mounted using DPX (Sigma-Aldrich).

Four sections from the whole/right hemisphere including the striatum, globus pallidus, substantia nigra, ventral hippocampus and cingulate cortex were scanned (digitized) on a LEICA SCN400F scanner to produce 20× magnification digital images. Depending on the region, 2–4 optical fields were taken, each comprising a 628.0 × 278.5 μm^2^ area. Ferritin-, GFAP- or Iba1-immunopositive cells in each optical field were manually counted using the cell counter plugin of ImageJ (NIH) and expressed as a number of immunopositive cells per unit area.

### Statistical Analysis

Data analysis was performed using Prism version 8 (GraphPad Software, CA, USA). Normal distribution was checked graphically using Q-Q plots, residual plot, homoscedasticity plot, and numerically using Shapiro-Wilk’s test. The following variables were log-transformed to normality: iron, zinc, copper, microglia, astrocytes, and ferritin. Analysis of variance (ANOVA) was used to determine differences between the different regions of the brain, and at different mouse brain ages. We also performed linear regression modeling to assess the association between metals and glia. Metals to glia ratio were computed to assess how the ratios changed relative to one another with aging. A statistical value of *p* ≤ 0.05 was considered significant. Values are quoted as mean ± standard deviation (SD).

## Results

### Qualitative Assessments

#### SRXRF Elemental Mapping of Metal Distributions in the Brain

**Figure 1:**
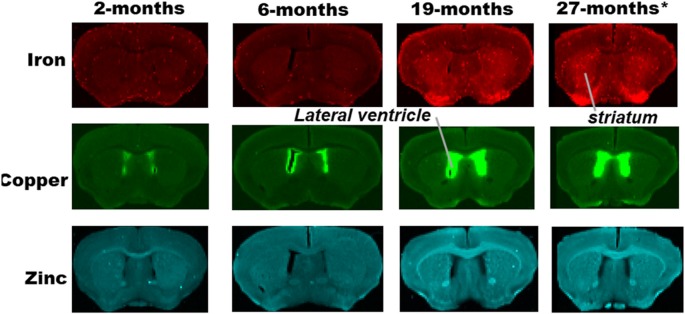
Synchrotron-radiation X-ray fluorescence (SRXRF) elemental iron, copper and zinc maps of tissue sections (30 μm thick) for quantification of the striatum of C57Bl/6J mice at 2, 6, 19 and 27-months of age. Other brain structures of interest are also labeled on the maps. High signal intensity artefacts arising from folded tissues and specks/pieces of tissue from cryosectioning are visible in some images.

**Figure 2:**
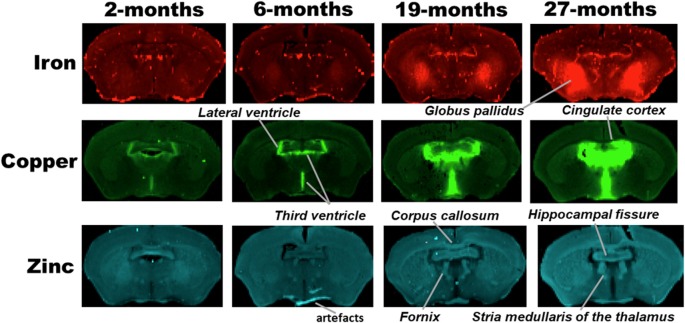
SRXRF elemental iron, copper and zinc maps of tissue sections (30 μm thick) for quantification of globus pallidus and cingulate cortex of C57Bl/6J mice at 2, 6, 19 and 27-months of age. Other brain structures of interest are also labeled on the maps. High signal intensity artefacts arising from folded tissues and specks/pieces of tissue from cryosectioning are visible in some images.

**Figure 3:**
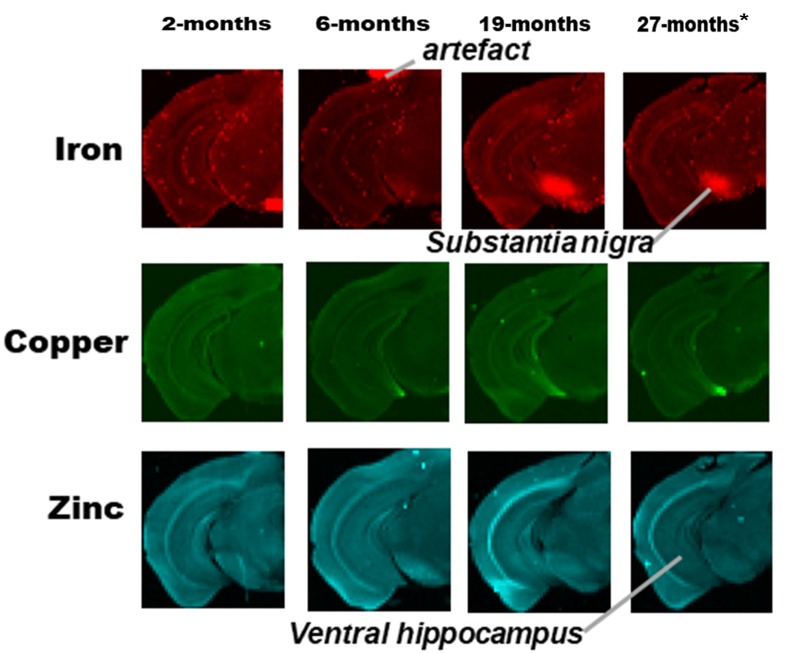
SRXRF elemental iron, copper and zinc maps of tissue sections (30 μm thick) for quantification of substantia nigra and ventral hippocampus of one hemisphere, of C57Bl/6J mice at 2, 6, 19 and 27-months of age. High signal intensity artifacts arising from folded tissues and specks/pieces of tissue from cryosectioning are visible in some images. High signal intensity artefacts arising from folded tissues and specks/pieces of tissue from cryosectioning are visible in some images. *The tissue sections collected from mice 27-month-old were not fully scanned by SRXRF to minimize scan time.

Metal distributions in the basal ganglia, cingulate cortex, and ventral hippocampus, were mapped with SRXRF and found to be both heterogeneous and altered with aging (Figures 1–3). Qualitative assessment of iron revealed increased deposition in the basal ganglia in 19- and 27-month-old mice, compared to the younger age-groups. Interestingly, the stria medullaris of the thalamus, fornix and the ventricles showed some qualitative iron increments at 19- and 27-months of age especially at the latter age (Figure 2).

Copper was strikingly enriched in the choroid plexus/ventricles at 19- and 27-months of age (Figure 2). Zinc, on the other hand, appeared generally elevated throughout the brain but particularly in the stria medullaris of the thalamus, the corpus callosum, fornix and hippocampal fissure at 19- and 27-months (Figure 2).

#### Immunohistochemistry of Glia Distribution

**Figure 4:**
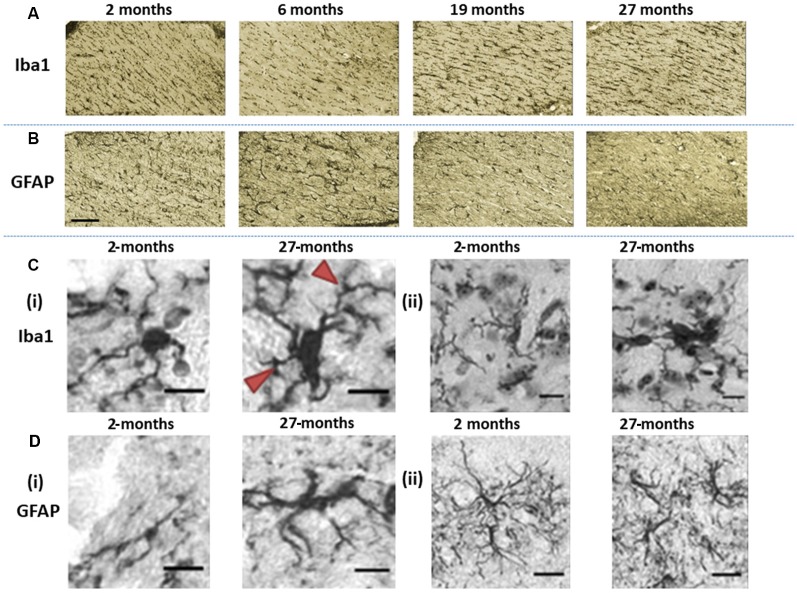
Immunohistochemically stained images of **(A)** Iba1-stained microglia and **(B)** glial fibrillary acidic protein (GFAP)-stained astroglia acquired at the level of the hippocampal fimbria and ventral commissure (20× magnification, scale bar = 100 μm). **(Ci)** The images show differences in Iba1-immunopositive microglia morphology in the basal ganglia, in 2-months old C57Bl/6J with microglia having small soma and ramified processes, compared to hypertrophic cell bodies and thickened processes (arrows) at 27-months of age (40× magnification, scale bar = 40 μm). **(Cii)** Fragmentation of processes is demonstrated by microglial staining in the basal ganglia of 27-month-old C57Bl/6J mice (40× magnification, scale bar = 40 μm). **(Di)** GFAP-stained astrocytes in 2-months old C57Bl/6J mice exhibit a small cell body with long, thin primary processes, while at 27-months of age, astrocytes show a spheroid cell body and thickened processes with a loss of distal processes (40× magnification, scale bar = 40 μm). **(Dii)** astrocytes in 2-month-old C57Bl6/J mice show manifold processes and therefore serve a greater territory, while those in 27-month-old mice exhibit loss of distal processes, de-ramified processes that have become shorter and thickened (40× magnification, scale bar = 40 μm).

Microglial (Iba1) immunostaining at the level of fimbria and ventral commissure appeared to be lower in 6-month-old mice compared to those at 2-month, but higher at ages 19- and 27-months relative to younger mice (Figure 4A). On the contrary, astrocytes (GFAP) seemed to show an age-related decline at the fimbria/ventral commissure level (Figure 4B).

Since age is routinely associated with glial dystrophy, we performed a qualitative evaluation of aged glia compared to younger glia in the basal ganglia (Figure 4). Aged microglia (27-month) exhibited signs of hypertrophy, with enlarged soma compared to 2-month-old mice in the substantia nigra and striatum (Figure 4Ci). The cell body of aged microglia exhibited a spheroid shape that was distinctive from the round shape of young microglia. Equally, the microglial processes observed in 27-month-old mice were shorter and thickened compared to the thinly ramified processes apparent in the 2-month old mice. Also, aged microglia commonly clustered together and was accompanied by frequent fragmentation of processes (Figure 4Cii).

Aged astrocytes exhibited hypertrophy, somata were larger and more elongated with processes appearing shorter and thicker, reminiscent of loss of distal processes (Figure 4Di). Younger astrocytes were marked by manifold long processes to serve a greater territory (Figure 4Dii).

### Quantitative Assessments

#### SRXRF Assessment of Brain Metals

##### Regional Brain Concentrations

ROIs were used to probe the metal concentrations in the basal ganglia, cingulate cortex and the ventral hippocampus (Supplementary Figure S1). Iron distribution was similar between different brain areas at age 2-month (Supplementary Figure S2i). However, at 6-months of age, significantly higher iron content was observed in the substantia nigra (*p* < 0.01) and globus pallidus (*p* < 0.05), compared to the cingulate cortex and ventral hippocampus. A similar trend was observed in 19- and 27-months old mice, where, significantly augmented iron concentrations were observed in the substantia nigra (*p* < 0.05 and *p* < 0.001, respectively) and globus pallidus (*p* < 0.001) compared to the striatum, cortex, and hippocampus.

The copper concentration was significantly lower in the globus pallidus compared to the striatum (*p* < 0.01), cingulate (*p* < 0.01) and ventral hippocampus (*p* < 0.05) at 6-months, but not observed at other ages (Supplementary Figure S2ii). Zinc levels were significantly elevated in the globus pallidus and hippocampus compared to the substantia nigra at 2-months of age (*p* < 0.05), but were lower in the globus pallidus compared to the nigra (*p* < 0.05) at 6-months (Supplementary Figure S2iii), with no changes observed at later ages.

##### Alterations of Regional Brain Metal Concentrations With Aging

**Figure 5:**
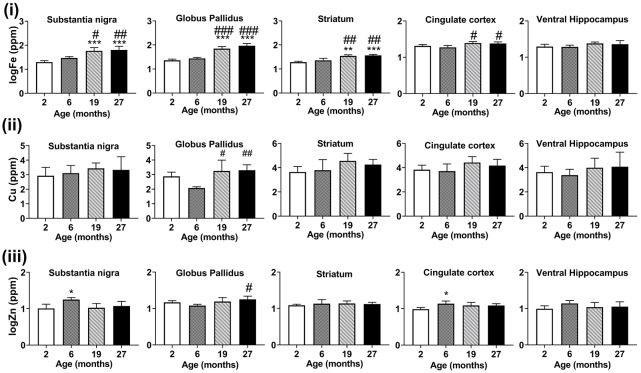
Graphs showing SRXRF measured changes in the levels of **(i)** elemental iron (Fe), **(ii)** copper (Cu), and **(iii)** zinc (Zn) in the substantia nigra, globus pallidus, striatum, cingulate cortex and ventral hippocampus with increasing age. Significance level at *p < 0.05; **p < 0.01 and ***p < 0.001, compared to 2-month, ^#^*p* < 0.05; ^##^*p* < 0.01; and ^###^*p* < 0.001, compared to 6-month. Iron and zinc were log-transformed. Values are mean ± standard deviation (SD; *n* = 4/age).

With advancing age, at ages 19- and 27-months, iron concentrations were significantly increased compared to 2- and 6-month-old mice (Figure 5i), in the substantia nigra (*p* < 0.001, *p* < 0.05, respectively), globus pallidus (*p* < 0.001) and striatum (*p* < 0.01, *p* < 0.001, respectively). In the cingulate cortex, iron-enrichment was observed in the 19- and 27-months old mice, compared to both 2- and 6-months old mice (*p* < 0.05), but were comparable between ages in the ventral hippocampus.

Higher levels of copper were found at 19-month (*p* < 0.05) and 27-month (*p* < 0.01) compared to 6-months old mice in the globus pallidus (Figure 5ii). Zinc levels were augmented in the 6-month-old mice compared to mice aged 2-months in the substantia nigra (*p* < 0.05) and cingulate cortex (*p* < 0.05; Figure 5iii). The globus pallidus exhibited increased zinc at aged 27-months (*p* < 0.05) compared to mice at 6-months.

#### Immunohistochemical Assessments of Ferritin, Microglia and Astrocytes in the Basal Ganglia

##### Regional Numbers of Ferritin-, Microglia- and Astrocyte-Immunopositive Cells

**Figure 6:**
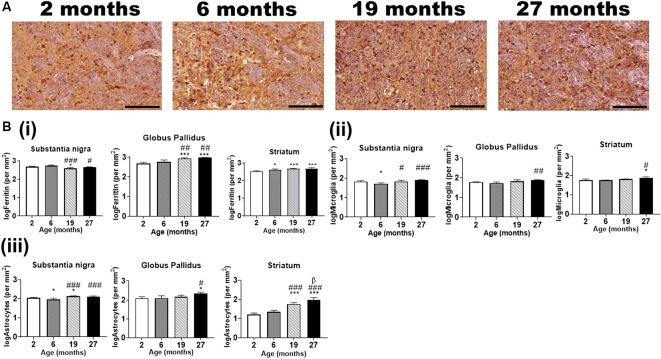
**(A)** Typical immunohistochemical stained images of ferritin in the globus pallidus with hematoxylin-eosin counterstaining (purple) of the cell nuclei at different ages. Scale bar: 100 μm. **(B)** Quantitative immunohistochemical analysis of **(i)** ferritin-, **(ii)** Iba1 (microglia)- and **(iii)** GFAP (astrocytes)-immunopositive cells in the basal ganglia in mice aged 2-, 6-, 19- and 27-months. Significance level at *p < 0.05 and ***p < 0.001, compared to 2-month; ^#^*p* < 0.05; ^##^*p* < 0.01; and ^###^*p* < 0.001, compared to 6-month and β, *p* < 0.05 compared to 19-months. Ferritin, Iba1, and GFAP data were log-transformed. Values are mean ± standard deviation (SD) (*n* = 4/age).

IHC revealed ferritin-immunoreactive cells (Figure 6A) to be significantly enriched in the substantia nigra and globus pallidus compared to the striatum at age 2-months (*p* < 0.01), however, only the globus pallidus (*p* < 0.05) had significantly more ferritin-immunopositive cells compared to the striatum at 6-months (Supplementary Figure 3i). A higher number of ferritin-immunoreactive cells (*p* < 0.001) was observed in the substantia nigra relative to both the globus pallidus and striatum at older ages (19- and 27-months). Also, the striatum demonstrated higher numbers of ferritin-immunoreactive cells compared to the globus pallidus at 19-months of age (*p* < 0.001).

Interestingly, IHC for Iba1 did not reveal significant differences in microglial positivity between the brain regions at different ages (Supplementary Figure S3ii). On the other hand, GFAP IHC revealed a consistent pattern of significant differences between the regions at all ages, with higher number of astrocytes in the globus pallidus compared to the substantia nigra (*p* < 0.001) and the striatum at ages 2-, 6- and 19-months (*p* < 0.001) and 27-months (*p* < 0.05; Supplementary Figure S3iii).

##### Alterations in Numbers of Ferritin- and Glial-Immunopositive Cells With Aging

Ferritin-immunopositive cells were less abundant in the substantia nigra at 19-months of age compared to that at 2- and 6-months (*p* < 0.05, *p* < 0.001, respectively), while the cell numbers at 27-months were lower relative to that at 6-months (*p* < 0.05) but not at 2-months (Figure 6Bi). On the contrary, the globus pallidus (*p* < 0.001) showed significantly enriched ferritin-immunopositive cell populations at 19- and 27-months compared to that at younger ages, 2- and 6-months. As expected, striatal ferritin-immunoreactive cells were augmented at aged 6-, 19- and 27-months relative to that at 2-months (*p* < 0.05, *p* < 0.001 and *p* < 0.001, respectively).

In the substantia nigra, microglial cells were found to be higher in the 2- (*p* < 0.05), 19- (*p* < 0.05) and 27-months old mice (*p* < 0.001) compared to that in the 6-months old (Figure 6Bii). The globus pallidus (*p* < 0.01) and striatum (*p* < 0.05) demonstrated a higher microglial-immunopositivity at aged 27-months compared to 6-months, while striatal microglial cells were also higher compared to 2-months (*p* < 0.05).

GFAP-immunostaining revealed significantly higher astrocytes in the 2-month and 19-month-old mice compared to that in the 6-month old, in the substantia nigra (Figure 6Biii). The astrocytes were observed to be augmented in the 19- and 27-month-old in relation to the 6-month-old mice. In the globus pallidus, astrocytes were significantly enriched at 27-month (*p* < 0.05) compared to both 2- and 6-month-old mice. Striatal astrocyte (*p* < 0.001) content was higher in the 19- and 27-month old compared to the 2- and 6-month old. Also, astrocytic staining was significantly higher at aged 27-month relative to 19-month (*p* < 0.05).

#### Relationships Between Cell-Immunoreactivities and Metals in the Basal Ganglia

##### Association of Brain Iron to Ferritin-Immunoreactivity

**Figure 7:**
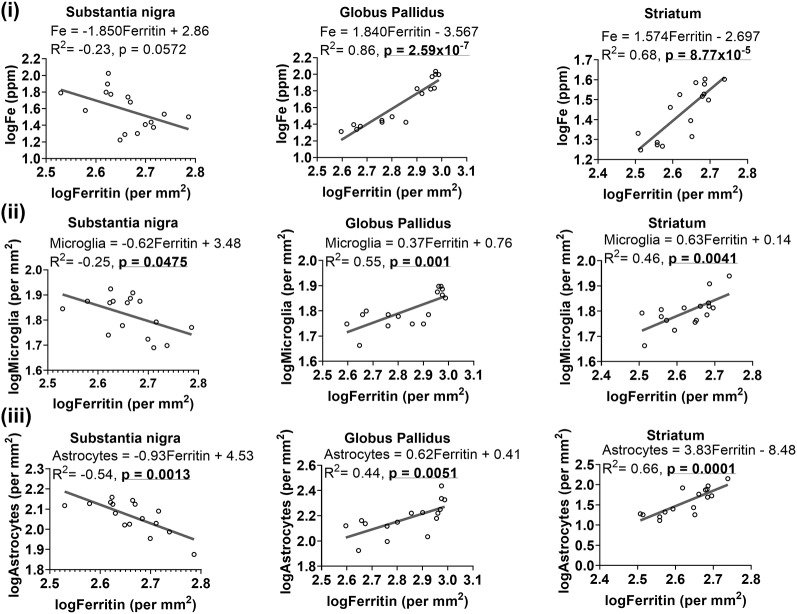
Linear regression analysis was performed to examine the association between ferritin with iron **(i)**, microglia **(ii)** and astrocytes **(iii)** in the basal ganglia. The regression equation for each graph, correlation coefficient (*R*^2^) and *p*-value (*n* = 16) for each analysis is noted.

As ferritin is an iron-sequestering protein, we correlated ferritin-immunoreactivity and iron levels. Higher iron levels were generally associated with attenuated levels of ferritin-immunoreactive cells in the substantia nigra, albeit significance at *p* < 0.05 was not reached (*R*^2^ = −0.23, *p* = 0.0572). On the contrary, in the globus pallidus (*R*^2^ = 0.86, *p* = 2.59 × 10^−7^) and striatum (*R*^2^ = 0.68, *p* = 8.77 × 10^−5^), ferritin levels were positively correlated to iron concentrations (Figure 7i).

We also formulated an iron to ferritin ratio to assess how both measures change in relation to one another in the basal ganglia with age. A significant increase in the iron:ferritin ratio (Supplementary Figure S4i) was observed at aged 19- and 27-months compared to 2- and 6-months, in the substantia nigra (*p* < 0.01, *p* < 0.05, respectively) and globus pallidus (*p* < 0.05, *p* < 0.01, *p* < 0.001, respectively). In the striatum, a higher iron: ferritin ratio was observed only at 27-months (*p* < 0.05) of age relative to 2- and 6-months.

##### Ferritin- and Glial-Immunoreactive Cells

Since glial cells predominantly contain light-chain ferritin, we correlated ferritin with Iba1 or GFAP immunoreactivities and found heterogeneity in associations between ferritin and glial cells in the basal ganglia (Figure 7). Higher levels of ferritin immunopositive cells were correlated to lower levels of microglia in the substantia nigra (*R*^2^ = −0.25, *p* = 0.0475), while a positive association was observed between ferritin and microglia in the globus pallidus (*R*^2^ = 0.55, *p* = 0.001) and striatum (*R*^2^ = 0.46, *p* = 0.0041; Figure 7ii). Interestingly, a similar trend was apparent for ferritin and astrocytes (Figure 7iii), where ferritin was negatively correlated to astrocytes in the substantia nigra (*R*^2^ = −0.54, *p* = 0.0013) but positively correlated to astrocytes in the globus pallidus (*R*^2^ = 0.44, *p* = 0.0051) and striatum (*R*^2^ = 0.66, *p* = 0.0001).

##### Relationship Between Metal Levels and Glia Numbers

**Figure 8:**
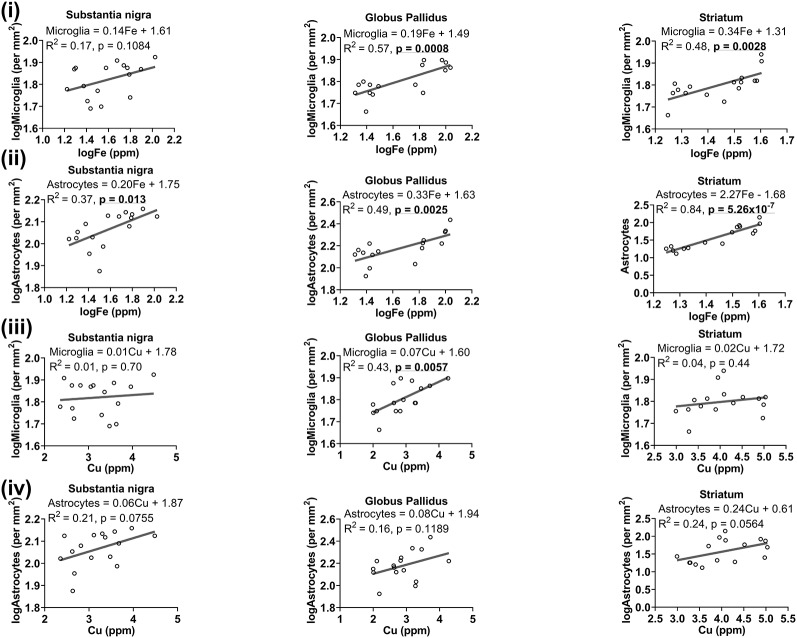
Linear regression analysis was performed to assess the association between **(i)** iron and microglia, **(ii)** iron and astrocytes, **(iii)** copper and microglia, **(iv)** copper and astrocytes in the basal ganglia. The regression equation for each graph, correlation coefficient (*R*^2^) and *p*-value (*n* = 16) for each analysis is noted.

We reasoned that glia, particularly those that surround neurons, may accumulate metals to maintain homeostasis locally to protect neurons from metal-induced oxidative stress, leading us to a plausible examination of the association between metal levels and glial cell numbers. Higher levels of iron were associated with higher number of microglia in the globus pallidus (*R*^2^ = 0.57, *p* = 0.0008) and striatum (*R*^2^ = 0.48, *p* = 0.0028), but not in the substantia nigra (*R*^2^ = 0.17, *p* = 0.1084; Figure 8i). A positive linear correlation (Figure 8ii) was observed between iron levels and astrocytes in substantia nigra (*R*^2^ = 0.37, *p* = 0.013), globus pallidus (*R*^2^ = 0.49, *p* = 0.0025) and striatum (*R*^2^ = 0.84, *p* = 5.26 × 10^−7^).

While generally, a higher concentration of copper was related to higher numbers of microglia in the basal ganglia, significance was only reached in the globus pallidus (*R*^2^ = 0.43, *p* = 0.0057), and not in other basal ganglia regions (Figure 8iii). There was also a generally positive association between copper and astrocytes in the substantia nigra (*R*^2^ = 0.21, *p* = 0.0755), globus pallidus (*R*^2^ = 0.16, *p* = 0.1189) and in the striatum (*R*^2^ = 0.24, *p* = 0.0564), albeit not significant at the *p* < 0.05 level (Figure 8iv).

**Figure 9:**
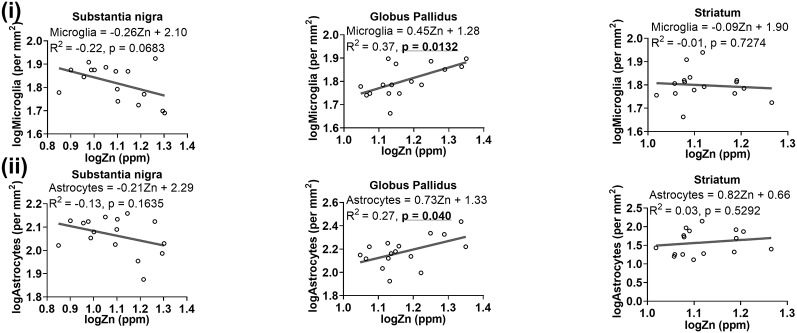
Linear regression analysis was performed to assess the association between **(i)** zinc and microglia, **(ii)** zinc and astrocytes in the basal ganglia. The regression equation for each graph, correlation coefficient (*R*^2^) and *p*-value (*n* = 16) for each analysis is noted.

A trend of negative relationship between zinc levels and microglia was apparent in the substantia nigra (*R*^2^ = −0.22, *p* = 0.0683), but a significant positive association was demonstrated in the globus pallidus (*R*^2^ = 0.37, *p* = 0.0132; Figure 9i). Moreover, a higher level of zinc was associated with higher numbers of astrocytes (*R*^2^ = 0.27, *p* = 0.040; Figure 9ii).

##### Aging-Associated Changes in Metal to Glia Ratio

Aging is associated with perturbed brain metal homeostasis and altered glial biology and therefore the aim of the present study was to evaluate how metal levels change in relation to the glia in the basal ganglia with advancing age. To investigate this, we calculated metal to glia ratios to elucidate the changing metal concentrations relative to glia (Supplementary Figure S4).

The iron:microglia ratio was augmented in the substantia nigra in 19- (*p* < 0.01) and 27-month (*p* < 0.05) old compared to that in 2-month-old mice (Supplementary Figure S4ii). Similarly, the globus pallidus demonstrated a higher iron:microglia ratio at aged 19- and 27-months, relative to that at 2-months (*p* < 0.001) and 6-months (*p* < 0.01, *p* < 0.001). The striatum also exhibited a higher iron:microglia ratio at aged 19- and 27-months compared to that at 2-months and a lower iron:microglia ratio was observed at 6-months compared to 19-months. The iron:astrocytes ratio was elevated in the substantia nigra and globus pallidus in the 19- (*p* < 0.05, *p* < 0.001, respectively) and 27-month-old mice (*p* < 0.001) compared to the 2-month-old (Supplementary Figure S4iii). Moreover, aged 19- (*p* < 0.001) and 27-month-old (*p* < 0.01) mice showed a higher iron:astrocytes ratio in the globus pallidus compared to in the younger 6-month-old mice. Meanwhile, the striatal iron:astrocytes ratio was found to be significantly lower in the older 19- and 27-months mice in relation to the younger aged 2 and 6-months old mice (*p* < 0.001, *p* < 0.05 and *p* < 0.001, *p* < 0.001, respectively).

Copper:microglia ratios were similar across the younger and older aged groups in the basal ganglia (Supplementary Figure S4iv), however, the copper:astrocytes ratio was attenuated at ages 19- and 27-month-old compared to their younger counterparts (Supplementary Figure S4v), 2-month (*p* < 0.001) and 6-month old (*p* < 0.01, *p* < 0.001). The substantia nigra exhibited increased zinc:microglia at 6-months vs. 2- (*p* < 0.01), 19- (*p* < 0.05) and 27-months (*p* < 0.05, Supplementary Figure S4vi), a similar pattern to that for the nigral zinc:astrocytes ratio (Supplementary Figure S4vii). An augmented striatal zinc:astrocytes profile was found in the 2-month old (*p* < 0.001) compared to the 6- and 19-month-old, while a lower zinc:astrocytes ratio was evident at 19- (*p* < 0.01) and 27-months (*p* < 0.001) compared to 6-months.

## Discussion

We demonstrate altered metal deposition and glia dystrophy in the basal ganglia regions with increasing age, which could explain the susceptibility of selected brain regions to neurodegenerative disease. Most noticeably, increased heterogeneity and differential distributions of the metals in the various brain regions with aging was apparent. Specifically, iron was highly enriched in the basal ganglia and exhibited age-related increases. Meanwhile, copper and zinc seemed to show modest increments in their concentration confined to the globus pallidus with aging, with zinc appearing to be enriched in nerve bundles. Moreover, levels of microglia and astrocytes increased as a function of age in the basal ganglia, but the aged brain showed dystrophic glia, reminiscent of senescence. Since aging is associated with a perturbed BCSFB, metal compositions were observed to be altered, particularly striking was copper deposition at the choroid plexus/ventricles. Taken together, we demonstrate that changes in brain barrier permeability and glial dystrophy with aging may induce differential regional content of various metals and are a hallmark of the aging brain.

### Iron

Consistent with previous reports including our own (Walker et al., 2016), iron was present at higher concentrations in the substantia nigra and globus pallidus compared to that in the striatum, cortex, and hippocampus at the older ages (Hallgren and Sourander, 1958). The iron levels increased with age predominantly in the basal ganglia, suggesting iron to be an age-dependent enriched metal in the brain (Connor et al., 1990). The augmented iron levels can be attributed to the constant delivery of iron into the brain and decreased release of iron into the blood *via* the BBB and BCSFB (Burdo and Connor, 2003). It is tempting to postulate that iron may be drained from the cerebrospinal fluid (CSF) *via* the lymphatic system (Ashraf et al., 2018). Interestingly, an *in vitro* study showed the transport of unbound iron from CSF to the blood *via* a DMT1-mediated transport mechanism (Wang et al., 2008). Furthermore, following *in vivo* exposure of rats to toxic amounts of manganese, the clearance of iron from the CSF to the blood was significantly diminished, leading to increased CSF iron deposition (Wang et al., 2008). Defective iron clearance as a function of aging may explain the qualitative increases in ventricular iron in older-aged mice we observed. Moreover, astrocytes which serve vital functions including transport of iron across brain-barrier systems and maintenance of brain iron homeostasis (Kubik and Philbert, 2015; Ashraf et al., 2018), demonstrated qualitatively age-related decreases in the regions close to the BCSFB (fimbria/ventral commissure level) in this study, reinforcing apparent impaired iron clearance from the brain with aging.

Usually, elevated iron is consistent with altered expression levels of iron-binding proteins, especially of ferritin, as its expression is induced by increased iron (Burdo et al., 1999). Indeed, increased ferritin mirrors the iron deposition in the globus pallidus and striatum we observed. However, a dissociation between iron accumulation and ferritin upregulation was observed in the aged substantia nigra, where higher levels of iron were not associated with increased levels of ferritin as we and others have reported previously (Benkovic and Connor, 1993; Walker et al., 2016). Ferritin is composed of 24 subunits of two types, heavy and light chains, forming a soluble hollow shell capable of storing up to 4,500 ferric iron atoms (Harrison and Arosio, 1996). This cellular iron storage protein is present in neurons, microglia, and oligodendrocytes, with oligodendrocytes being the highest iron-containing cell in the brain (Benkovic and Connor, 1993). Interestingly, the detection of ferritin in astrocytes is unusual, in that astrocytes exhibit weak ferritin immunoreactivity (Mirza et al., 2000). Heavy chain ferritin exhibits ferroxidase activity and converts the reactive toxic ferrous ion to the more stable ferric ion, so that iron can be stored by light chain ferritin (Muhoberac and Vidal, 2013). Microglia predominantly contain light chain ferritin as they are more concerned with scavenging iron (Ashraf et al., 2018) and have been shown to accumulate more iron than neurons, with microglia being better scavengers than astrocytes (Bishop et al., 2011). In the present study, we found a positive linear correlation between ferritin- and glial cell-immunoreactive cells in the globus pallidus and striatum, consistent with a previous report (Schipper et al., 1998). Interestingly, ferritin immunopositive microglia has been demonstrated to become more pronounced with age, whereas neuronal ferritin staining remains unaltered despite elevated iron as assessed by Perl’s histochemical staining (Benkovic and Connor, 1993). Elevated iron in neurons, in the absence of a concomitant increase in neuronal ferritin, may predispose neurons to iron-induced free radical damage and ensuing oxidative stress. In the vulnerable aged brain, the role of glial ferritin in sequestrating and detoxifying iron is even more paramount.

Negative correlation was observed between ferritin- and GFAP-immunopositive cells in the substantia nigra. Due to its exceptionally high iron content, the substantia nigra adopts two different iron storage systems, one based on ferritin and the other, on neuromelanin (Tribl et al., 2009), enabling synergistic regulation of iron homeostasis. However, our age-related observation of decreased ferritin along with reports signifying declining neuromelanin content (Tribl et al., 2009; Xing et al., 2018) in the aged substantia nigra, suggest an increased ferrous iron pool that can precipitate oxidative stress during brain aging, contributing to the increased risk of neurodegenerative diseases, particularly PD, with aging (Dexter et al., 1987; Xu et al., 2018).

We and others have previously reported increased microglial and astroglial cell numbers with aging in the basal ganglia, providing evidence for a more primed or inflammatory profile (Codazzi et al., 2015; Walker et al., 2016; Boisvert et al., 2018). Aging is associated with chronically elevated levels of circulating cytokines including TNFα, IL1β and TGFβ, with glial cells being major driving factors for brain aging (von Bernhardi et al., 2010). Interestingly, microglial and astroglial iron homeostasis has been shown to be differentially regulated by TNFα and TGFβ. Treatment of astrocytes with pro-inflammatory TNFα induced expression of DMT1 and suppressed ferroportin expression, while anti-inflammatory TGFβ-treatment did not affect DMT1 expression but increased ferroportin expression (Rathore et al., 2012). On the contrary, treatment of microglia with either TNFα or TGFβ leads to augmented DMT1 expression together with suppression of ferroportin. The findings demonstrate that TNFα leads to iron uptake and retention by both microglia and astrocytes, while TGFβ promotes iron efflux from astrocytes but increased microglial iron retention (Rathore et al., 2012). Astrocytes appear to be amenable to modulation, while microglia are fuelled by iron to perpetuate inflammation, hastening the aging process and contributing to increased susceptibility to acquiring the neurodegenerative disease.

Interestingly, we found an increased iron to microglia ratio in the basal ganglia, while the iron to astroglia ratio was elevated in the substantia nigra and globus pallidus but decreased in the striatum during aging. This could be indicative of the different roles performed by astrocytes on a regional basis *per se*. The nigral astrocytes have been found to be overly sensitive to acute ischemia compared to other brain regions (Karunasinghe et al., 2018), and exhibit vulnerability to oxidative insult (Cardoso et al., 2012, 2014). The aged nigra is associated with both iron and copper deposition in subsets of astrocytes (Schipper et al., 1998), rendering these astrocytes inherently prone to perturbations in metal redox homeostasis. Oxidative inactivation of aconitase, aka iron-responsive protein-1 (IRP1), which is involved in regulating cellular iron, in astrocytes has been linked to increased ferrous iron and hydrogen peroxide production (Cantu et al., 2009), promoting bioactivation of dopamine and other catechols to neurotoxic free radicals. Of note, augmented release of IL-1β and TNFα by iron-loaded microglia induced upregulation of IRP1, DMT1, hepcidin and transferrin receptor-1 (TfR1) expression in ventral mesencephalic neurons *via* production of reactive oxygen species (ROS), enhancing neuronal iron accumulation (Xu et al., 2016). Inefficient sequestration of redox-active iron by aging nigral glia, concomitant with the dissociation between iron and ferritin upregulation mentioned above, may predispose the senescent nervous system to oxidative stress-mediated neurodegeneration (*via* Fenton reactions) in Parkinsonism and other neurodegenerative diseases (Zecca et al., 2004).

While microglia may be unequivocally involved in perpetuating the generation of ROS in the striatum, astrocytes may play more of a neuromodulatory role (Pelizzoni et al., 2013). The declining iron to astroglia ratio in the striatum observed in the present study could be indicative of a compensatory response mediated by astrocytes to regulate iron levels (Knott et al., 1999). Striatal astrocytes are commissioned with the task of physiological clearance of age-related synaptic debris, particularly of degenerated dopaminergic neurons (Morales et al., 2017). Dopaminergic neurons undergo an insidious degeneration (~6–8% of cells every decade) during normal aging while in PD, a loss of 60% of striatal synapses is evident (Rodriguez et al., 2015). Previous data suggests that if astrocytes can perform functional trans-autophagy of cell debris, this should be sufficient to ensure striatal tissue homeostasis. However, the onset of PD may be characterized by impaired astroglial trans-autophagy, necessitating microglial activation to complete the clearance of dopaminergic neuronal debris, thereby aggravating the onset and progression of PD (Morales et al., 2017).

As previously documented, microglia and astroglia demonstrated pronounced morphological changes with aging in the basal ganglia, where senescent dystrophic glia exhibited hypertrophic soma and decreased arborization of processes (Jyothi et al., 2015). The glial cells may undergo aberrant signaling, leading to age-related metal dyshomeostasis, and represent a concause in neurodegenerative processes.

### Copper

Copper levels were higher in the striatum, cingulate cortex, and ventral hippocampus compared to the globus pallidus in 6-month-old mice. The older mice exhibited increases in copper levels in the globus pallidus compared to 2- and 6-month-old mice, as well as high copper levels, were correlated with an augmented number of microglia. Akin to iron, copper can also undergo Fenton chemistry and so high copper levels promote ROS production, and altered copper homeostasis is prevalent in neurodegenerative diseases (Wang et al., 2010; Zheng et al., 2010; Zheng and Monnot, 2012). Aging is characterized by a chronic neuroinflammatory state and interferon γ-stimulated microglial cells have been associated with augmented copper uptake accompanied by an expression of the copper importer, copper transporter-1 (CTR1) particularly in the choroid plexus (Zheng et al., 2010). Copper was strikingly enriched in the ventricles with aging in the present study, consistent with previous observations of increased copper at the choroid plexus (Fu et al., 2015). It has been suggested that the BCSFB is the predominant barrier for regulated copper uptake in the brain (Choi and Zheng, 2009). Astrocytes in the vicinity of ventricles are in a prime location to balance brain copper content and achieve detoxification, having access to both interstitial fluid and CSF (Pushkar et al., 2013). Moreover, the concentrations of other metals did not reach the striking levels exhibited by copper with aging, implying that astrocytes are chiefly involved in regulating brain copper levels. Our finding of increased ventricular copper deposition is further strengthened by literature showing augmented genetic and protein expression levels of copper transporters (e.g., ATP7A) at the choroid plexus compared to the brain parenchyma (Choi and Zheng, 2009; Fu et al., 2015). We found attenuated astrocytic expression close to the ventricles at the level of ventral commissure/fimbria with aging and concomitant with astrocytes being intimately involved in sequestrating copper, age-associated dysregulation of astroglial copper-buffering may occur and lead to toxic copper accumulation (Zheng and Monnot, 2012). As increased copper levels have been linked to reduced neurogenesis (Pushkar et al., 2013) which may be another contributory factor to the susceptibility of the aged brain to neurodegenerative diseases.

### Zinc

Zinc is a pleiotropic modulator of synaptic plasticity, neuronal activity, and cognitive processes, with ~30% of zinc in the brain existing as the free/chelatable form and stored within synaptic vesicles of glutamatergic forebrain neurons and the remaining ~70%, in proteins (Portbury and Adlard, 2017). Zinc levels in the brain are regulated by metallothioneins, zinc- and iron-like regulatory proteins (ZIPs) and zinc transporter proteins (ZnTs; Hennigar and Kelleher, 2012). Zinc is released from presynaptic vesicles into the synaptic cleft, coincident with glutamate and regulates e.g., long-term potentiation *via* activation of N-methyl-D-aspartate (NMDA) receptors (Takeda and Tamano, 2012). Histochemical staining visualizes free/chelatable zinc, usually synaptic zinc, has long-established zinc to be highly localized in nerve bundles and subsequently proved by proton-induced X-ray emission spectroscopy (Danscher et al., 1985). Similarly, we observed zinc to be enriched in the stria medullaris of the thalamus, corpus collosum, the fornix, and the hippocampal fissure, especially in older aged brains. Further, zinc accumulation had been shown to be higher in the hippocampus compared to other brain regions with the exception of the cerebellum following the administration of radioactive zinc to rats (Sawashita et al., 1997). This is consistent with the higher zinc in the ventral hippocampus compared to the substantia nigra at 6-months of age that we observed and underscores the importance of zinc in learning and memory consolidation (Sindreu and Storm, 2011). Zinc levels were also higher in the globus pallidus than in the substantia nigra at 2-months of age, albeit comparable to that in the hippocampus. Zinc has been known to modulate GABAergic transmission in the globus pallidus (Chen and Yung, 2006) and zinc is likely to have a major role during neurodevelopment in this brain region. However, the globus pallidus demonstrated lower zinc levels compared to the substantia nigra at 6-months of age. Indirect inputs from the striatal medium spiny neurons to the substantia nigra pars recta are *via* the external part of the globus pallidus and mediated by inhibitory neurons such that medium spiny neurons excitation further inhibits the thalamus *via* this pathway (Frank, 2005). The reversal in the zinc gradient between the globus pallidus and the substantia nigra as mice matured from 2 to 6 months of age suggests alterations in electrical activity/tone of this indirect basal ganglia circuit during neurodevelopment. We also observed a similar pattern of increased zinc in the cingulate cortex as mice aged from 2 to 6 months of age, consistent with zinc-containing neurons being required in brain development to form complex and elaborate associational network to interconnect the cortex with the limbic system (Corona et al., 2011).

Brain zinc levels have been reported to be increased with aging in both rats (Sawashita et al., 1997) and in man (Markesbery et al., 1984). We also observed augmented zinc levels in the globus pallidus of 27-months old mice compared to 2-, 6- and 19-months old mice. Interestingly, zinc accumulation has been reported in the globus pallidus in the 6-hydroxydopamine rat model of PD (Tarohda et al., 2005) and in the substantia nigra and striatum of PD subjects (Dexter et al., 1989). Thus, the augmented zinc in the globus pallidus of very old mice suggests zinc dysregulation may be another factor in aging being the major risk factor for neurodegenerative disease. While we did not observe significantly increased hippocampal zinc levels, this has been observed in aging rats and zinc chelation therapy was shown to attenuate deficits in synaptic plasticity (Shetty et al., 2017). Note, the discrepancy may arise as SRXRF maps elemental zinc content, rather than free/chelatable zinc, which is often the form detected by other zinc measurement techniques.

Zinc has primarily been known to be associated with neuronal function, however, glial cells are commissioned with the task of maintaining zinc homeostasis, thereby maintaining optimal synaptic signaling (Hancock et al., 2014). We found a significant association between zinc and glial cells in the globus pallidus. Indeed, astrocytes accumulate zinc (Nolte et al., 2004) *via* expression of the zinc transporter, ZIP14 (Bishop et al., 2010), enabling astrocytes to maintain glutamatergic and GABAergic synaptic transmission (Hancock et al., 2014). Also, microglia can directly uptake zinc *via* another zinc transporter, ZIP1, which serves as a trigger for sequential microglial activation (Higashi et al., 2011). The glial senescence observed in this study may induce aberrant connectivity at the tri-partite synapse by enhancing zinc dyshomeostasis with aging.

During neurodevelopment (from 2 to 6 months of age), the increased zinc to glial ratio observed may reflect increased availability of zinc to neurons and glia for synaptogenesis/synaptic pruning. In humans, peak synaptic density has been demonstrated to occur in mid-childhood (Liu et al., 2012), with synaptic pruning extending to a third decade of life (Petanjek et al., 2011). However, the ratio of zinc to astroglia was reduced in the basal ganglia at the older ages which may be attributed to the age-associated astrogliosis observed. Thus, a situation of functional zinc deficiency may be induced, as opposed to zinc excess as suggested above, regardless, either scenario may contribute to the synaptic dysfunction observed in aging (Szewczyk, 2013).

## Conclusion

Metal dyshomeostasis and the presence of a low-grade chronic inflammation owing to dystrophic glial cells are pervasive features of normal aging, rendering the brain susceptible to neurodegenerative diseases.

## Data Availability Statement

According to UK research councils, Common Principles on Data Policy, data supporting this study will be openly available in the Supplementary Material.

## Ethics Statement

Ethical review and approval was not required for the animal study because brain samples were purchased/received, the authors did not have live animals at any time.

## Author Contributions

P-WS contributed to the conception and design of the study. AE, TW, MS, AS, and SA performed the histology and data analysis. P-WS, CM, HP, AP, TW, and KG acquired and analyzed SRXRF data. AA performed the statistical data analysis. P-WS and AA wrote the first draft of the manuscript with contributions from AE, MS, and TW. AA, P-WS, AE, HP, MS, and SA revised and approved the submitted version.

## Conflict of Interest

KG is employed by Diamond Light Source. The remaining authors declare that the research was conducted in the absence of any commercial or financial relationships that could be construed as a potential conflict of interest.
