# Supplementary material for: Regional Distributions of Iron, Copper and Zinc and Their Relationships With Glia in a Normal Aging Mouse Model
Source: Front Aging Neurosci. 2019 Dec 19;11:351. doi: 10.3389/fnagi.2019.00351 (PMC6930884; doi:10.3389/fnagi.2019.00351)
Supplement: Supplementary file 2 [file Data_Sheet_1.pdf]

Regional distributions of iron, copper and zinc and their relationships with glia in a normal aging mouse model

Azhaar Ashraf[1], Christos Michaelides[1], Thomas A. Walker[1], Antigoni Ekonomou[1], Maria Suessmilch[1], Achvini Sriskanthanathan[1], Semhar Abraha[1], Adam Parkes[1], Harold G Parkes[1], Kalotina Geraki[2], Po-Wah So*[1]

[1]Department of Neuroimaging, Institute of Psychiatry, Psychology and Neuroscience, King's College London, London, United Kingdom, [2]Diamond Light Source, Harwell Science and Innovation Campus, Didcot, Oxfordshire, United Kingdom.

***Address for correspondence**:

Dr Po-Wah So

Department of Neuroimaging,

Institute of Psychiatry, Psychology and Neuroscience,

King's College London,

Maurice Wohl Clinical Neuroscience Institute,

5, Cutcombe Road,

Denmark Hill Campus,

London. SE5 9RX

UK

Running title: Metals in aging mouse brain

Keywords: synchrotron radiation-based X-ray fluorescence elemental mapping, microglia, astrocytes, iron, copper, zinc, aging, glial dystrophy

Supplementary figures

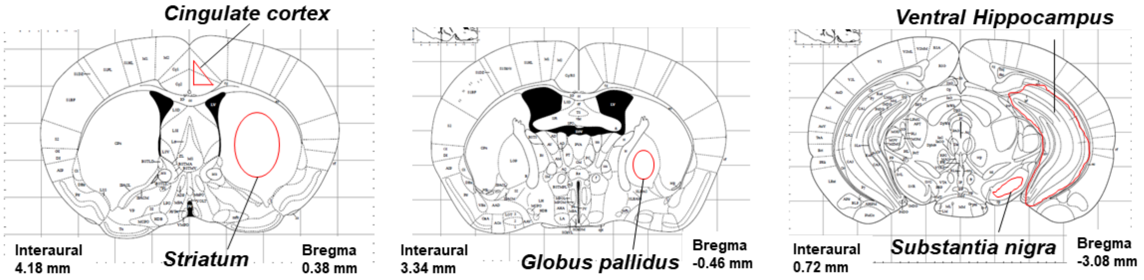

Figure 1: Representative ROIs (red lines) for the measurement of metal concentrations in synchrotron-radiation X-ray fluorescence (SRXRF)-derived elemental maps in the mouse brain. Figures are adopted from the mouse brain atlas (Paxinos, G. and Watson, C. (1986) The Rat Brain in Stereotaxic Coordinates. Academic Press, New York).

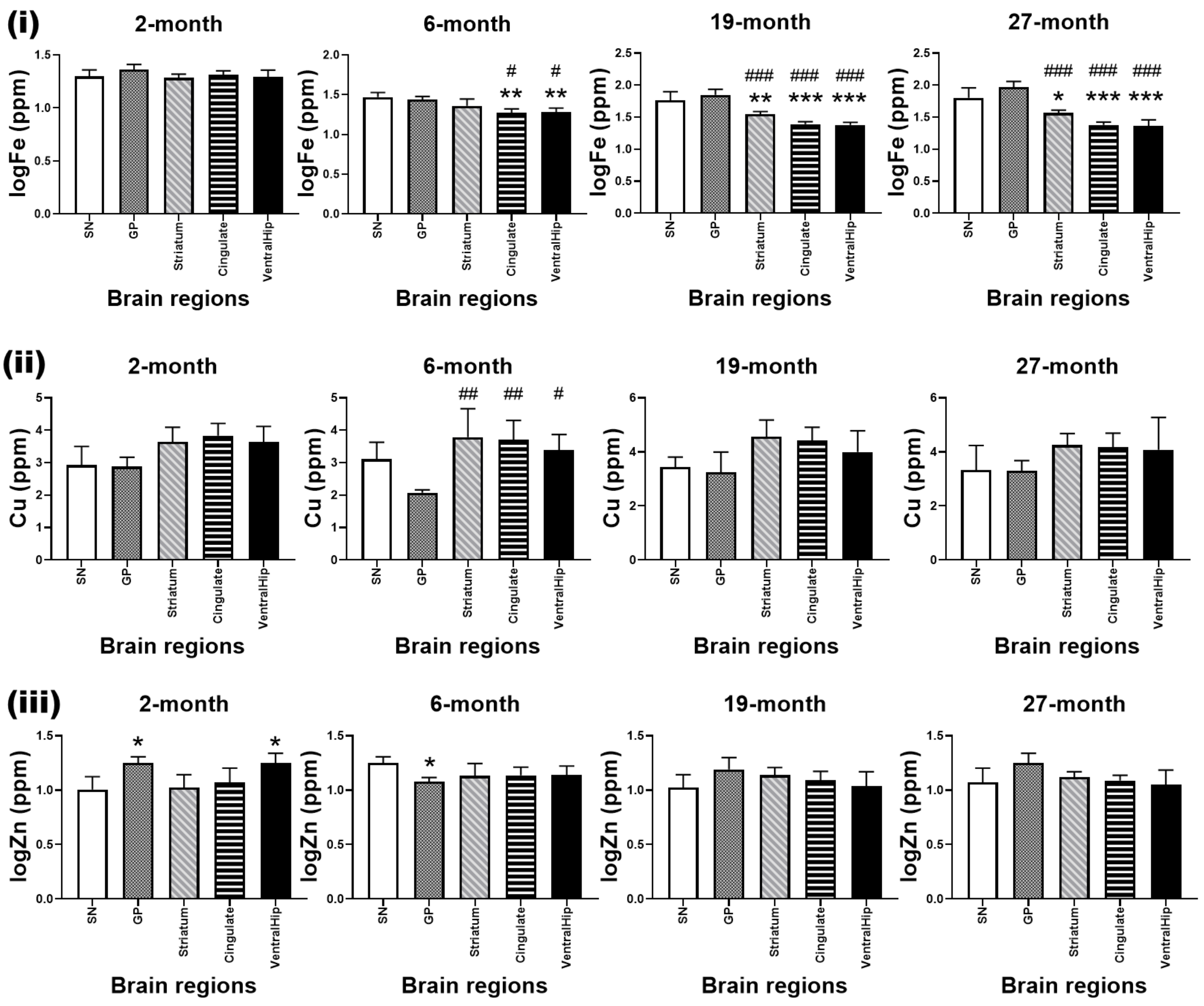

Figure 2: Synchrotron-radiation X-ray fluorescence measured levels of (i) elemental iron (Fe), (ii) copper (Cu), and (iii) zinc (Zn) in the substantia nigra (SN), globus pallidus (GP), striatum, cingulate cortex and ventral hippocampus (VentralHip) from mice aged 2-, 6-, 19- and 27-months. Significance level at *, $p<0.05$; **, $p<0.01$ and ***, $p<0.001$, compared to SN; #, $p<0.05$; ##, $p<0.01$; and ###, $p<0.001$, compared to GP. Iron and zinc were log-transformed. Values are mean ± standard deviation in parts per million (ppm).

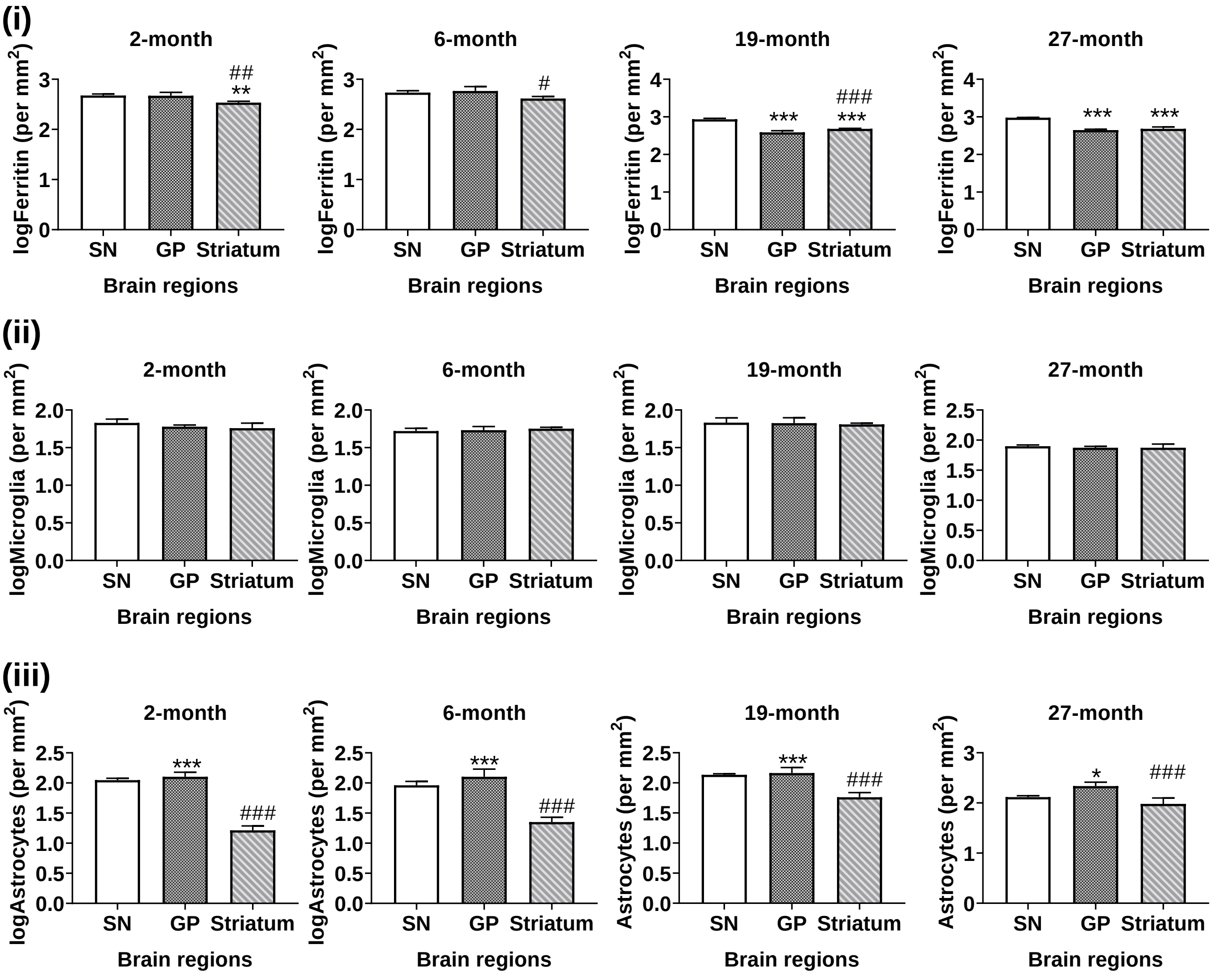

Figure 3: Alterations in (i) ferritin-, (ii) microglia- and (iii) astrocyte-immunopositive cells in the substantia nigra (SN), globus pallidus (GP) and striatum from mice aged 2-, 6-, 19- and 27-months. Significance level at *, $p<0.05$; **, $p<0.01$ and ***, $p<0.001$, compared to SN and ###, $p<0.001$, compared to GP. Ferritin, microglia and astrocytes data were log-transformed. Values are mean ± standard deviation.

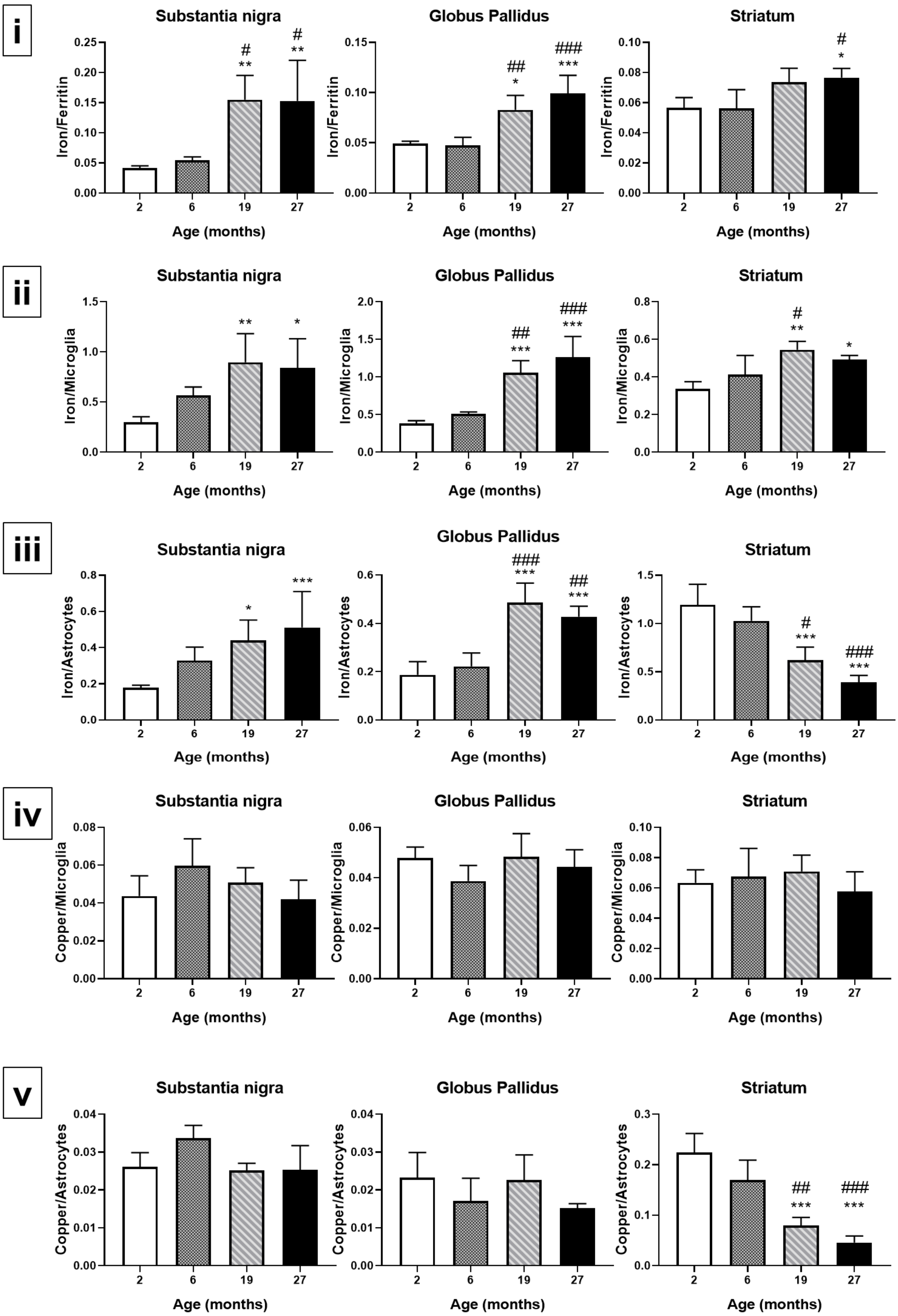
i
Substantia nigra
Globus Pallidus
Striatum
Iron/Ferritin
ii
Substantia nigra
Globus Pallidus
Striatum
Iron/Microglia
iii
Substantia nigra
Globus Pallidus
Striatum
Iron/Astrocytes
iv
Substantia nigra
Globus Pallidus
Striatum
Copper/Microglia
v
Substantia nigra
Globus Pallidus
Striatum
Copper/Astrocytes
Age (months)
2
6
19
27

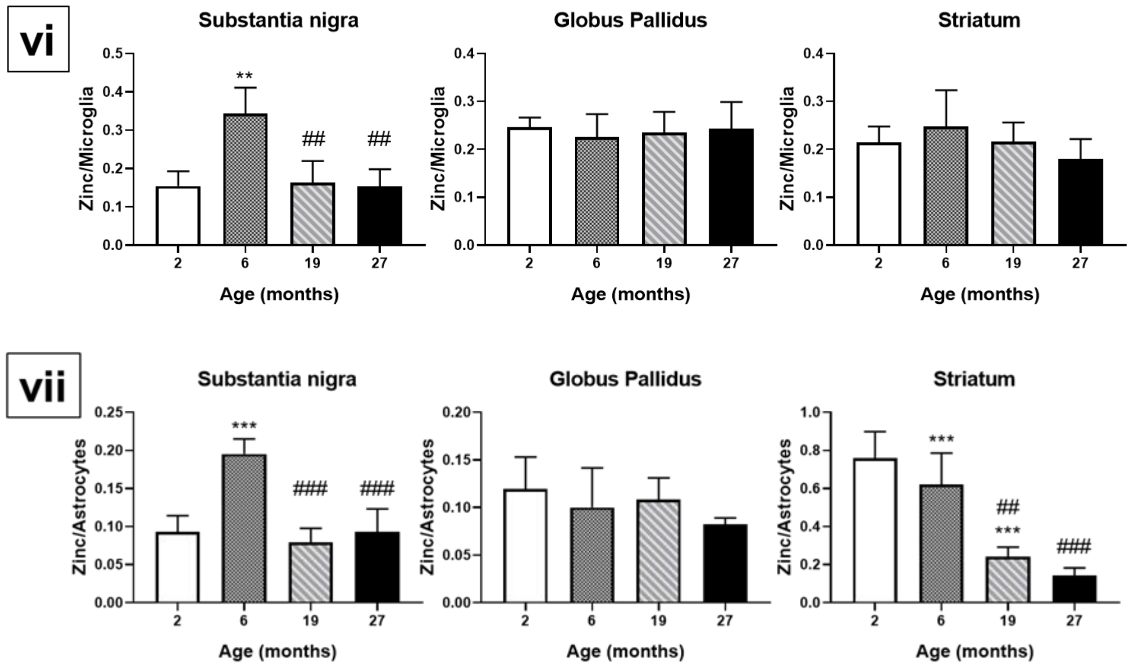

Figure 4: Ratios of (i) elemental iron (Fe):ferritin, (ii) Fe:microglia, (iii) Fe:astrocytes, (iv) copper (Cu):microglia, (v) Cu:astrocytes, (vi) zinc (Zn):microglia, and (vii) Zn:astrocytes, in different basal ganglia regions of mice aged 2-, 6-, 19- and 27-months. Significance level at *, $p<0.05$; **, $p<0.01$ and ***, $p<0.001$, compared to 2-month; #, $p<0.05$; ##, $p<0.01$; and ###, $p<0.001$, compared to 6-month. Values are mean ± standard deviation.
